# Mid-troposphere transport of Middle-East dust over the Arabian Sea and its effect on rainwater composition and sensitive ecosystems over India

**DOI:** 10.1038/s41598-017-13652-1

**Published:** 2017-10-20

**Authors:** V. Ramaswamy, P. M. Muraleedharan, C. Prakash Babu

**Affiliations:** CSIR-National Institute of Oceanography, Goa, 403004 India

## Abstract

The importance of mineral dust and aerosols in the transfer of bio-essential elements to terrestrial and marine ecosystems far removed from the source region is well known. Aerosol concentrations measured at the surface over the west coast of India during the SW monsoon period (June to September) are usually very low as pristine maritime air from the Southern Indian Ocean blows over this region. However, we find very high levels of mineral dust and dust derived nutrients in rainwater collected during the SW monsoon period. We show that the dry, warm and dusty Red Sea Wind and Shamal Wind from the Middle-East override the moist oceanic Low-Level Jet (Findlater Jet) of the SW monsoon and transport large quantities of dust at heights between 2 km and 5 km over the Indian Peninsula. A substantial portion is the desert dust is scavenged and wet-deposited over the Western Ghats of India where it neutralizes the acidity of rainwater and provides substantial amounts of nutrients that have the potential to impact sensitive ecosystems in this region. After the Red Sea and Shamal Winds subside in September, the alkaline rainwater reverts to the acidic range due to soluble ions derived from local carbonaceous aerosols.

## Introduction

Mineral dust plays an important role in climate forcing by altering the radiative balance of the earth^1^. Mineral dust concentrations over the Middle-East have been shown to affect monsoon precipitation over India^2^. Long range transport of mineral dust affects ecosystems located far away from the source region^3–12^. For example, dust from the Sahara Desert affects the health of corals in the Caribbean^3–5^ and supplies essential nutrients^8,11,12^ to the Amazon rainforests. Mineral dust and aerosols also affect ocean primary productivity^4,13–15^ and biogeochemical processes, particularly through the supply of the micro-nutrient iron^10,13,14^.

**Figure 1:**
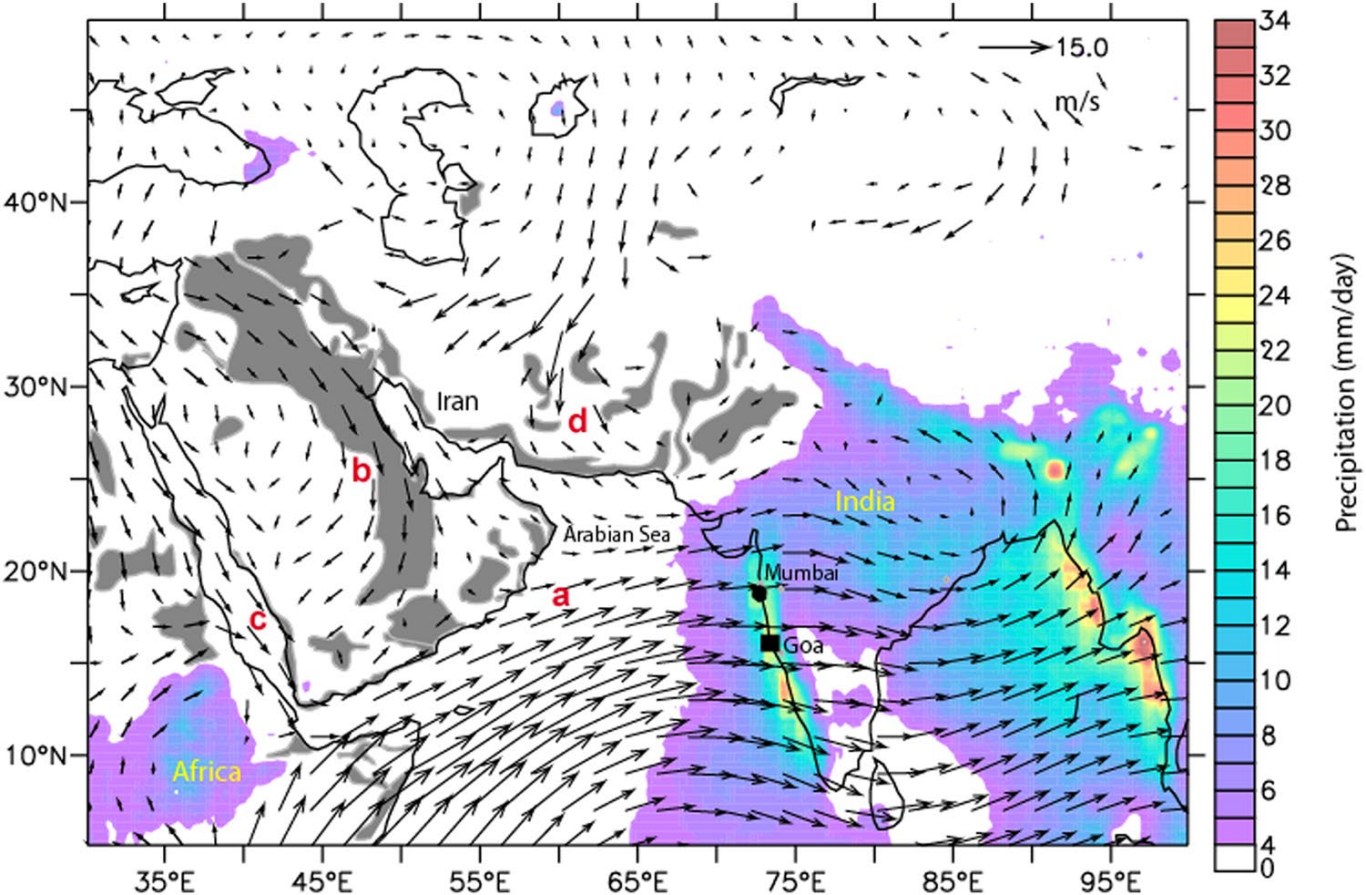
Quasi-climatology (2003–2014) of the major winds (850 hpa) over the northern Indian Ocean during summer monsoon period. The major wind systems are the (**a**) Low-Level Jet (Findlater Jet), (**b**) the Shamal winds over the Arabian Peninsula, (**c**) the Red Sea Winds, and (**d**) the northerly Levar Winds over SW Asia. Major dust source regions providing mineral dust to the northern Indian Ocean are shown in grey. Colour scale indicates average precipitation (2003–2014) for the month of June, July and August derived from the Tropical Rainfall Measuring Mission satellite. Non coloured areas (white or grey) indicate rainfall less than 4 mm per day. The figure has been created using open-source software Ferret version 6.73 available at http://ferret.pmel.noaa.gov/Ferret/downloads. MERRA wind data used in this study/project was downloaded in 2014 through the NASA GES DISC online archive http://wiki.seas.harvard.edu/geos-chem/index.php/MERRA provided by the Global Modeling and Assimilation Office (GMAO) at NASA Goddard Space Flight Center.

**Figure 2:**
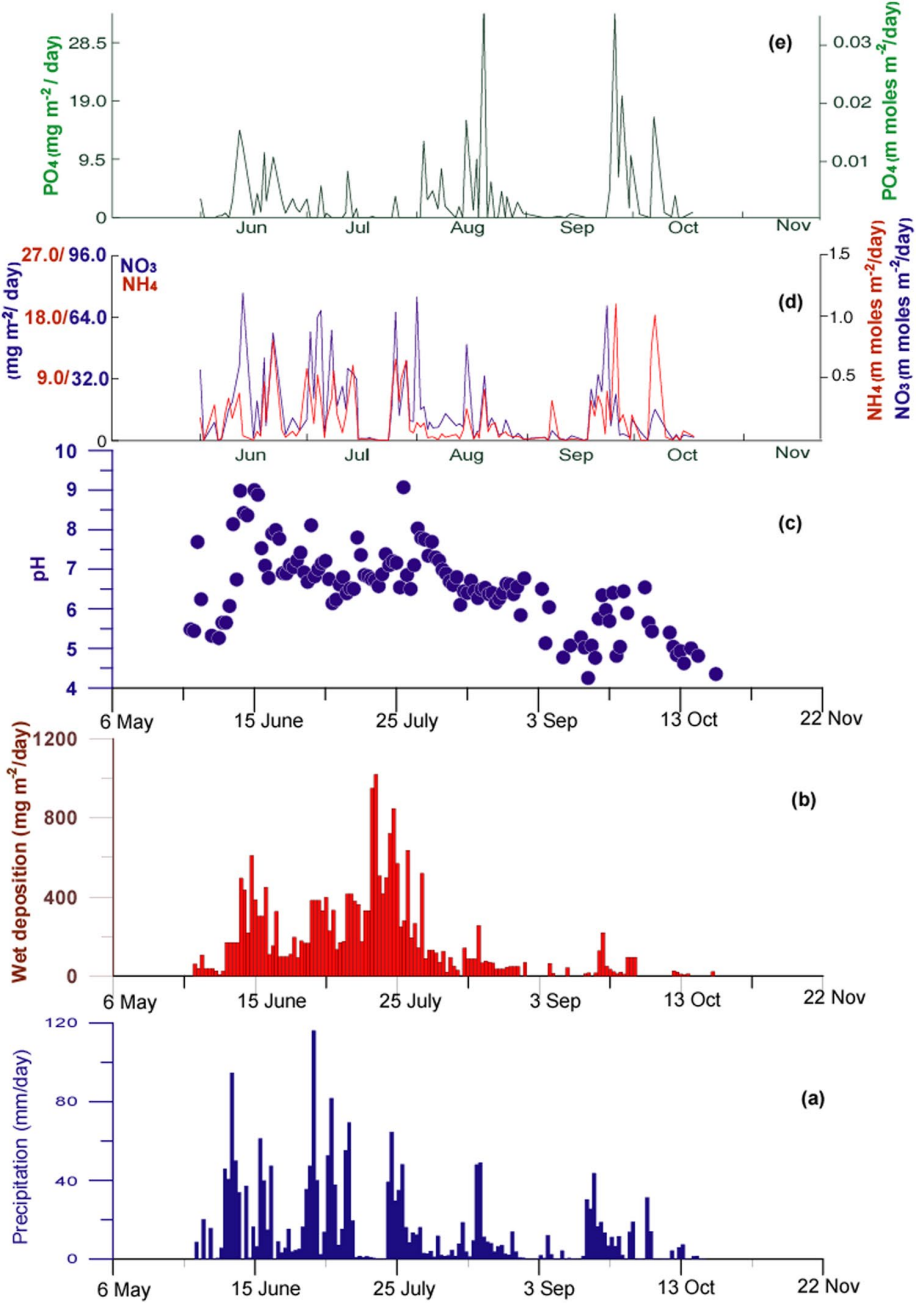
(**a**) Daily precipitation during 2013 (mm d^−1^) over Goa Station. There were no detectable rainfall events over Goa during 2013 other than shown in this figure. (**b**) Bulk wet deposition of aerosols (mg m^−2^ d^−1^). (**c**) pH of rainwater (**d**) Daily bulk wet deposition of dissolved NH_4_ and NO_3_ and (**e**) total dissolved PO_4_.

The summer heating over NW India and Tibet leads to convergence of winds from the surrounding high-pressure regions (Fig. 1) of the Indian Ocean, eastern Mediterranean and Central Asia. The summer monsoon winds which blow at the surface have very low aerosol content as they are derived mostly from pristine maritime regions in the Indian Ocean. Ship-based studies in the western and central Arabian Sea^16,17^ and coastal stations along the west coast of India (Supplementary information Fig. 1) report low aerosols at the surface during June to September. However, during the same season we measured very high levels of mineral dust and dissolved nutrients in rainwater (Fig. 2; Table 1) at a station (Goa, 15° 27′N; 73° 48′E) on the west coast of India. Earlier studies have indicated that dust may be transported above the marine boundary layer and wet deposition may play a significant role in their removal from the marine boundary layer^17,18^. In this study we discuss the mechanism of transport of Middle-East and African dust over the Arabian Sea and their effect on rainwater composition and sensitive ecosystems over the Indian Peninsula.

**Table 1:** Total wet deposition of major ions for the year 2013 in precipitation.

Ion	Wet Deposition	
	(millimoles m^−2^ y^−1^)	mg m^−2^ y^−1^
Na^+^	353.7	8131
NH_4_^+^	21.7	391
K^+^	9.11	356
Mg^2+^	44.4	1079
Ca^2+^	120.1	4813
Non Sea Salt Ca^2+^	105.9	4244
Cl^−^	427.5	15156
NO_3_−	31.3	1941
SO_4_^2−^	49.2	4726
PO_4_^3−^	0.4	38
Non Sea Salt SO_4_	28.4	2728
Ca/Na	0.34	0.59
N (NO_3_+NH_4_+30% PON)	34.15	478

After collection, rainwater was immediately filtered through a syringe Teflon filter (0.2 µm). Major cations and anions in rainwater were measured with a reagent free ion chromatograph (DIONEX 5000).

**Figure 3:**
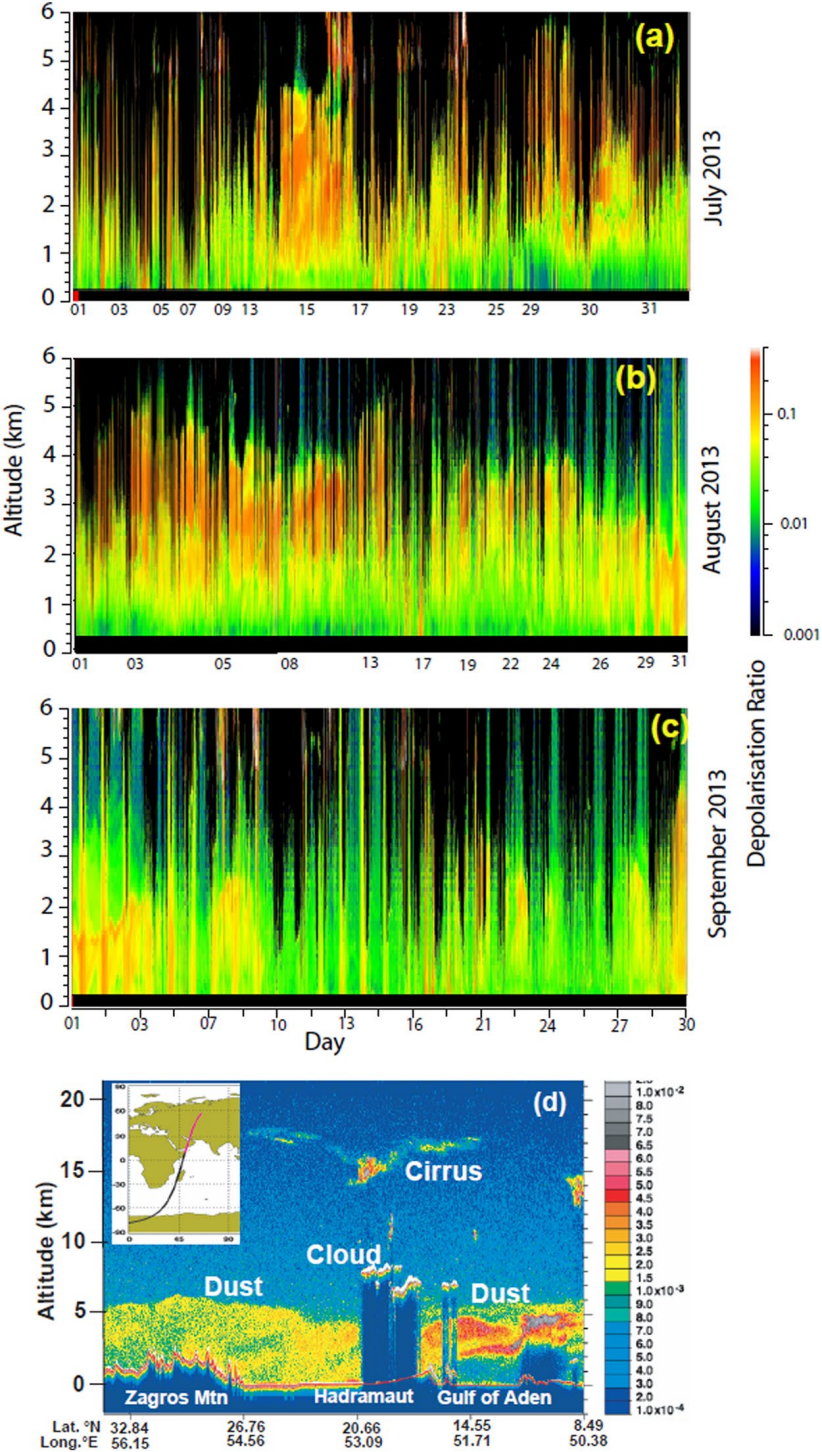
Multi Pulse LiDAR derived depolarization ratios over Goa, India for the months of (**a**) July (**b**) August and (**c**) September 2013. In July and August a dusty layer (depolarization ratios >0.1), is seen between 2 and 5 km. High depolarisation ratios above 6 km are due to icy cirrus clouds. Satellite LiDAR total attenuated backscatter profiles (Calipso) data over the western Arabian Sea (**d**) shows a dusty layer extending 5 km above mean sea level. A low aerosol layer at (0 to 2 km) is overlain by a high aerosol layer 2 to 5 km) in the Horn of Africa and Gulf of Aden. MPLiDAR data collected at Goa using a Sigmaspace MPL LiDAR and data processed and figure generated using SIGMAMPL software (Build version 2013.0.0.252; http://sigmaspace.com). CALIPSO image of 10 July 2013was obtained from the NASA Langley Research Center Atmospheric Science Data Center (https://www-calipso.larc.nasa.gov/products/lidar/browse_images/show_calendar.php.).

We first demonstrate the interaction of contrasting air masses of oceanic and continental origin in the northwestern Arabian Sea as they advance towards the west coast of India. Evolution of the zonal wind from the western to the eastern Arabian Sea along 15°N, inferred from superimposed positive and negative anomalies of virtual potential temperature (VPT) (Supplementary information Fig. 2a–d), indicate convergence of these distinct air masses around 55°E–60°E. The eastward flowing Shamal and Red Sea Winds (RSW) bring in a 5 km thick dust layer from the Nubian Desert and Arabian Peninsula towards the Gulf of Aden and Western Arabian Sea (Fig. 3d). The powerful Low Level Jet penetrates into the lower part of this dusty layer in the western Arabian Sea pushing the dusty layer aloft (Fig. 3d). Due to the buoyancy difference, the hot continental wind Red Sea and Shamal winds override the LLJ (Supplementary information Fig. 2c) and forms an extended core as it approaches the west coast of India (Supplementary information Fig. 2d). HYSPLIT wind back-trajectories also indicate the convergence of different wind systems over the western Arabian Sea and over-riding of the LLJ by the dry and dusty continental air masses (Supplementary information Fig. 3). The time-series Vaisala Radiosonde data of temperature lapse-rate over Goa confirm a heterogeneous system, where warm and dry air pockets with low lapse-rate are present above a clean layer of oceanic air mass (Supplementary information Fig. 4a,b). Humidity profiles (Supplementary information Fig. 4b) also indicate presence of dry continental air mass above the humid maritime air mass. The temperature inversion prevents rainfall over the western and central Arabian Sea^19^. The air masses on reaching the Western Ghats along the west coast of India undergo orographic uplift resulting in intense rainfall^20,21^.

The presence of the dusty layer aloft is further confirmed by concomitant Micro-Pulse LIDAR time-series data over Goa (Fig. 3a–c) which indicates that the upper layer between 2 and 5 km are dominated by non-spherical mineral dust particles while the planetary boundary layer has very low aerosol content. The LiDAR records also show that most of the heavy rain spells over Goa coast originates from clouds located at heights less than 2.5 km. Scavenging by hydrometeors leads to the wet deposition of the mineral dust. Since the dusty layer extends to 5 km above mean sea level, only part of the dust is scavenged and a significant portion of the dust is transported up to the eastern Bay of Bengal (Supplementary Fig. 5). The heavy precipitation in the eastern Arabian Sea (Fig. 1) can remove most of the particles in the boundary layer before the air masses reach the Indian Peninsula. Satellite LiDAR data (Fig. 3d) shows that the marine boundary layer of the arid western and central Arabian Sea have lower dust concentrations and the bulk of the mineral dust is transported above the boundary layer. HYSPLIT models (Supplementary Fig. 3) and Satellite LiDAR data (Fig. 3d) indicate that the overriding of the continental air mass over the LLJ takes place in the western Arabian Sea close to Arabian coast.

We measured the average annual wet deposition of mineral dust over Goa (average of 4 years) as 20.7 g m^−2^. Maximum wet deposition of dust is observed during June-July when the summer Shamal winds are most active (Fig. 2b). The wet deposition rates decrease substantially after August after the Red Sea and Shamal Winds recede (Fig. 2b). Major rain events after September have negligible dust concentrations. The annual deposition rate of mineral dust over Goa is about 2 to 5 times higher than those measured by sediment traps over the western and central Arabian Sea^22,23^ as wet deposition is more efficient in removing atmospheric aerosols compared to gravity dependent dry deposition.

Chemical analysis of rainwater particulates (Supplementary information Table 1) shows high SiO_2_ (>58%) and Al_2_O_3_ (18%) indicating them to be mainly of crustal origin. The wet deposited particles over Goa bear a closer resemblance to soils derived from the Middle-East and NE Africa rather than local sources. Bulk mineral analysis of rain-water particulates by X-Ray diffraction (Supplementary Fig. 6) shows abundant Palygorskite, a mineral typically found in the Sabkhas of Arabia. Palygorskite/Illite ratios are similar to those found in the western Arabian Sea adjacent to the Yemen-Oman coast^24,25^. High palygorskite and smectite concentrations in aerosols along with satellite data and wind back trajectories (Supplementary Fig. 3) indicate the Saudi Arabian Peninsula and NE Africa as the main source of dust to the Arabian Sea and the west coast of India during the SW monsoon period^24–27^.

We now show that the interaction of mineral dust with rainwater leads to the addition of large amounts of soluble compounds to rainwater (Table 1), and neutralization of acid precipitation over Goa (Fig. 2c). The pH of rainwater is normally close to 5.6 due to absorption of carbon dioxide from the atmosphere. The pH of rainwater at Goa (Fig. 2c, Supplementary information Table 2) exhibit large variations between 9 and 4, being markedly alkaline during the SW monsoon but reverting to the acid range after September. Other studies have reported alkaline rain over the Indian Peninsula from stations located close to the core of the Low-Level Jet whereas stations located south of the LLJ have precipitation mainly in the acid range^28–31^. The high Ca/Na ratios in our rainwater samples (Table 1) indicate a major contribution from soils normally found in desert soils. The persistent high pH over Goa between June and August indicate a continuous supply of alkaline dust which cannot be from local acidic soils or aerosols. We therefore conclude that alkaline rain over the Indian Peninsula during the SW monsoon is mainly due to mineral dust derived from the Middle-East and NE Africa. After the Shamal Winds recede, the pH of rainwater changes to acidic range due to non-availability of dust for neutralization and contribution of acidic compounds like oxides of sulfur and nitrogen (Table 1) derived from combustion of biomass and fossil fuels^31,32^ from local sources. Rainwater samples from June to August are enriched with Non-Sea Salt Ca^2+^ (Table 1; Supplementary information Table 3) derived from desert dust while those in September and October are enriched in non-sea salt SO_4_^2+^, NO_3_^1−^, Cl^1−^ and K^1−^ derived mostly from carbonaceous aerosols. The rapid change in pH of rainwater and the end of the SW monsoon can affect extremely sensitive micro-ecosystem located in the Western Ghat Biodiversity Hotspot, like for example the precipitation fed rock pools or rainwater dependant epifauna^33^.

Annual deposition of soluble P in rainwater over Goa is about 100 g P ha^−1^ a^−1^. To estimate the particulate phosphorus flux associated with mineral dust deposition we need to know the P content is dust. We measured P_2_O_5_ content in bulk samples using X-Ray fluorescence (Supplementary information Table 1). Using a minimum phosphorus concentration of 0.08% (P_2_O_5_ in wet deposited material is 0.26% (Supplementary data Table 1). The proportion of P in P_2_O_5_ is 43.63%. P concentration is mineral dust should therefore be 0.1134%. Because of variation in P content of aerosols due to size segregation during transport we have taken a minimum value of P in wet deposited material as 0.08% and average wet deposition of dust as 20.7 g m^−2^ a^−1^ we estimate P deposition of 16.6 mg P m^−2^ a^−1^ which is equivalent to 166 g P ha^−1^ a^−1^. Numerous studies have shown that Sahara dust maintains the fertility of the Amazon rainforest^3,11,12^ particularly through the supply of phosphorus^34^ in mineral dust, which has recently been estimated^12^ to be about 23 (7–39) g P ha^−1^ a^−1^. In an earlier study Swap *et al*.^11^, have estimated Sahara dust derived P flux in the central Amazon as 11–47 g P ha^−1^ a^−1^. The annual deposition of dust derived P over Goa is about a magnitude larger than that of the Amazon rain forest. Though most of the P in rainforest are recycled^35^, mineral dust derived P can potentially meet about 4 percent of the total requirement of phosphorus in the Western Ghat rainforest and can therefore provide an important outside source of P that can compensate the hydrological loss of phosphorus on decadal time-scales in these P-depleted soils^36^ and help maintain the fertility of the Western Ghats rainforest. Similarly, the dust-laden nutrient rich precipitation can contribute substantially to coastal primary productivity by providing additional soluble nutrients^37,38^ like P and N (~100 g P ha^−1^ a^−1^and 6463 g N ha^−1^ a^−1^). Intensification of seasonal coastal anoxia along the west coast of India in recent years could be in part due to increasing dust fluxes sourced from the drying lakes of SW Asia^39^. However, further detailed studies are required to understand these processes.

## Conclusion

This study investigated the transport of mineral dust from the Middle-East and NE Africa over the Arabian Sea during the summer monsoon and its effect on rainwater pH and composition over Goa, India. Reanalysis data, Radiosonde data, LiDar data and satellite images show that the dry, warm and dusty Red Sea Wind and Shamal Wind from NE Africa and the Middle-East override the moist oceanic Low-Level Jet (Findlater Jet) of the SW monsoon and transport dust at heights between 2 km and 5 km over the Arabian Sea up to Indian Peninsula. The heavy precipitation along the eastern Arabian Sea and west coast of India scavenges and wet deposits about 20.7 g m^−2^ of dust annually. Mineralogy and chemistry of the wet deposited particles indicate them to of crustal origin derived from NE Africa and the Arabian Peninsula. The alkaline desert dust neutralizes the pH of rainwater over Goa. Rainwater pH from June to end of August is mostly in the alkaline range after which it falls in the acid range. We estimate the annual wet deposition of particulate P, dissolved P and dissolved N as 166 g P ha^−1^ a^−1^, 100 g P ha^−1^ a^−1^ and 6463 g N ha^−1^ a^−1^ respectively. The effects of change in pH of rainwater and wet deposition of nutrient elements on sensitive environments along the west coast of India need to be investigated in detail.

## Data and Methods

### Wind pathways in the northern Arabian Sea

To demonstrate the dynamics of major wind fields in the northern Arabian Sea, monthly mean wind, humidity, and temperature at 32 levels were extracted from ERA-interim (ECMWF reanalysis data) gridded data product during the peak summer month (July) of 2013. The zonal wind cross sections across various longitudes from west to east (40E, 50E, 60E and 70E) displayed the convergence of two contrasting air masses of oceanic [Low-Level Jet (LLJ)] and continental [Summer Shamal Wind (SSW)] origin as they advance to the west coast of India. To delineate the movement of air masses, the virtual potential temperature anomaly (${{\boldsymbol{\theta }}}_{{\boldsymbol{v}}}$) is computed and overlaid on zonal wind component. The ${{\boldsymbol{\theta }}}_{{\boldsymbol{v}}}$ is defined as the theoretical potential temperature of dry air that would have the same density as moist air and is derived using the following relationship.

$${{\boldsymbol{\theta }}}_{{\boldsymbol{v}}}=\,{\boldsymbol{\theta }}(1+0.61{{\boldsymbol{q}}}_{{\boldsymbol{v}}}-{{\boldsymbol{q}}}_{{\boldsymbol{l}}})$$

where ${\boldsymbol{\theta }}$ is potential temperature, ${{\boldsymbol{q}}}_{{\boldsymbol{v}}}$is the specific humidity of water vapor, and ${{\boldsymbol{q}}}_{{\boldsymbol{l}}}$ is the liquid water specific humidity. Here ${{\boldsymbol{q}}}_{{\boldsymbol{l}}}$ is set to zero for the calculations, as it is poorly observed and tends to be small when averaged over the time and space scales studied here.

It can serve as a stability criterion for an atmosphere with a moisture gradient. Since water vapor is less dense than dry air, humid air has warmer ${{\boldsymbol{\theta }}}_{{\boldsymbol{v}}}$ than dry air at the same pressure and temperature. But over Arabian Sea temperature effect often override the moisture effect and hence –ve and +ve ${{\boldsymbol{\theta }}}_{{\boldsymbol{v}}}$ anomaly (deviation from longitudinal average at respective height) signifies moist and dry air respectively. Increasing (decreasing) ${{\boldsymbol{\theta }}}_{{\boldsymbol{v}}}$ with altitude prescribe stable (unstable) condition of the atmosphere. The superimposed ${{\boldsymbol{\theta }}}_{{\boldsymbol{v}}}$ anomaly unambiguously demarcates these air masses by virtue of their contrasting characteristics

### Radiosonde data time series

The state of the art VaisalaDigiCORA III sounding system is used to launch RS-92 radiosondes for profiling the troposphere to collect once in three days time series (June to September) from the coastal station at the west coast of India. This radiosonde type features a GPS receiver for wind finding. It also has a silicon pressure sensor, heated twin humidity sensor and a small, fast temperature sensor. The dry bias correction due to solar heating is not called for as the local ascending time (1730 hrs) is closer to the sunset.All the RS-92 radiosondes were calibrated and procured 18 months before the campaign. Temperature lapse rate (100 m) and virtual potential temperature were also calculated to highlight effectively the mixing of contrasting air masses and eventual formation of a two layer system.

### Micro Pulse LiDAR (MPL) and Calipso satellite LiDAR Data

The Sigma Space Micro Pulse Lidar was operated at 532 nm with a power of 6.7 µJ@2500 Hz and the intensity of backscattered light was measured using photon-counting detectors using software provided by Sigma Space. The MPL is mounted inside an environment protection unit and was operated even during the rainy season. The Lidar derived time series of backscatter and extinction coefficient and depolarization ratio exhibits the vertical structure of spherical and non-spherical particles all through the troposphere during clear sky conditions. The extinction coefficient is a measure of the amount of aerosols in the atmosphere while the depolarization ratio is a measure of sphericity of the aerosols. Hydrometeors and carbonaceous aerosols have deploarization ratios less than 0.08 dust and mineral dust particles have deploarization ratios more than 0.08.

Satellite LiDAR browse images total attenuated backscatter profiles, an indicator of the concentration of particles in the atmosphere, were downloaded from Calipso site. (https://www-calipso.larc.nasa.gov/products/lidar/browse_images/show_calendar.php). Similar to MPL LiDAR depolarisation ratios were used to distinguish between spherical and non-spherical particles.

### Satellite images and data

Satellite data and visualizations used in this study were downloaded from the Giovanni online data system, developed and maintained by the NASA GES DISC. We also downloaded and studied satellite images and products from EUMETSAT, CALIPSO, KALPNA and INSAT 3A satellites.

Land Aerosol Optical Depth 550 nm (Deep Blue, Land-only) and corresponding Deep Blue Angstrom Exponent for land (0.412–0.47 micron, MYD08_D3 v6) from MODIS Aqua satellite were downloaded from the NASA Giovanni Site (https://giovanni.gsfc.nasa.gov/) at one degree grid for the period 2004 to 2014. Aerosol optical depth (AOD) is a measure of the extinction of the solar beam by particles in the atmosphere. The Angstrom exponent, ¨ α, is a qualitative indicator of aerosol particle with lower values indicating coarser particle size. Dust sources in Fig. 1 were broadly identified by plotting bimonthly quasi-climatology (average monthly dust AOD 2004–2014) of “dust AOD” over the entire region. Dust AOD is obtained when corresponding AE values are below a threshold (0.6) indicating the presence of mostly coarse mineral aerosols.

Monthly precipitation gridded data for 2003–2014 downloaded at grid interval of 0.25 degrees were downloaded from the NASA Tropical Rainfall Measuring Mission (TRMM) site (https://trmm.gsfc.nasa.gov/) and processed to prepare a quasi-climatology precipitation map.

Wind data were downloaded from the NASA MERRA site (https://gmao.gsfc.nasa.gov/reanalysis/MERRA/) for the period 2003–2014 and processed to obtain a detailed quasi-climatology map. MERRA is a NASA reanalysis product using the Goddard Earth Observing System Data Assimilation System Version 5 (GEOS-5).

### Precipitation collection and analysis

Rainwater was collected at the National Institute of Oceanography campus, Goa, India, about 500 m from the Arabian Sea. Major ions were measured on filtered rainwater with reagent free ion chromatography (DIONEX-5000). Ph was measured with a calibrated pH meter.

An autonomous weather station (AWS) provided wind (Anemometer) and precipitation (tipping bucket rain gauge) data (http://inet.nio.org/DonaPaula_AWS/) at 10-min intervals.

### Chemical and mineralogical analysis of wet deposited material

Particulate matter in rainwater was filtered using 0.4-micron polycarbonate filters and analyzed for mineralogy by XRD (Rigaku), major metal oxides were measured with a XRF (Phillips) and energy dispersive X-ray (Jeol scanning electron microscope).

### Data availability

The authors declare the main data supporting the findings of this study are available within this article and its supplementary material.

## Electronic supplementary material

Supplementary Information
