# Supplementary material for: Mid-troposphere transport of Middle-East dust over the Arabian Sea and its effect on rainwater composition and sensitive ecosystems over India
Source: Sci Rep. 2017 Oct 20;7:13676. doi: 10.1038/s41598-017-13652-1 (PMC5651843; doi:10.1038/s41598-017-13652-1)
Supplement: Supplementary file 1 — Supplementary Information [file 41598_2017_13652_MOESM1_ESM.pdf]

Mid-troposphere transport of Middle-East dust over the Arabian Sea and its effect on rainwater composition and sensitive ecosystems over India.

V. Ramaswamy*, P. M. Muraleedharan, C. Prakash Babu
CSIR-National Institute of Oceanography, Goa, India
Corresponding author e-mail: rams.goa@gmail.com; rams@nio.org

Supplementary data and figures

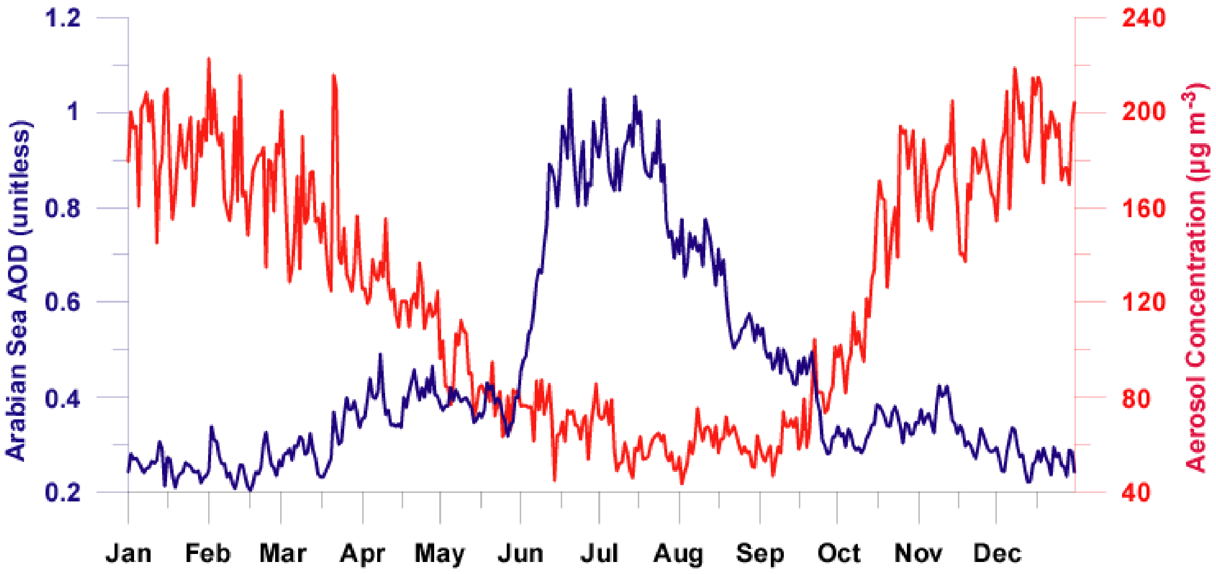

Supplementary Information Figure 1. Average Aerosol concentration (2007-2015) over Bandra, Mumbai on the west coast of India and Aerosol Optical Density (AOD) over the Arabian Sea. Highest satellite derived AOD values are during the SW monsoon period (June to September) while ground measurement at Mumbai during the same period shows the lowest aerosol concentrations. Satellite AOD data is from moderate-resolution imaging spectroradiometer (average of Terra and Aqua satellites) while aerosol concentration data is from the Maharashtra State Pollution Control Board.

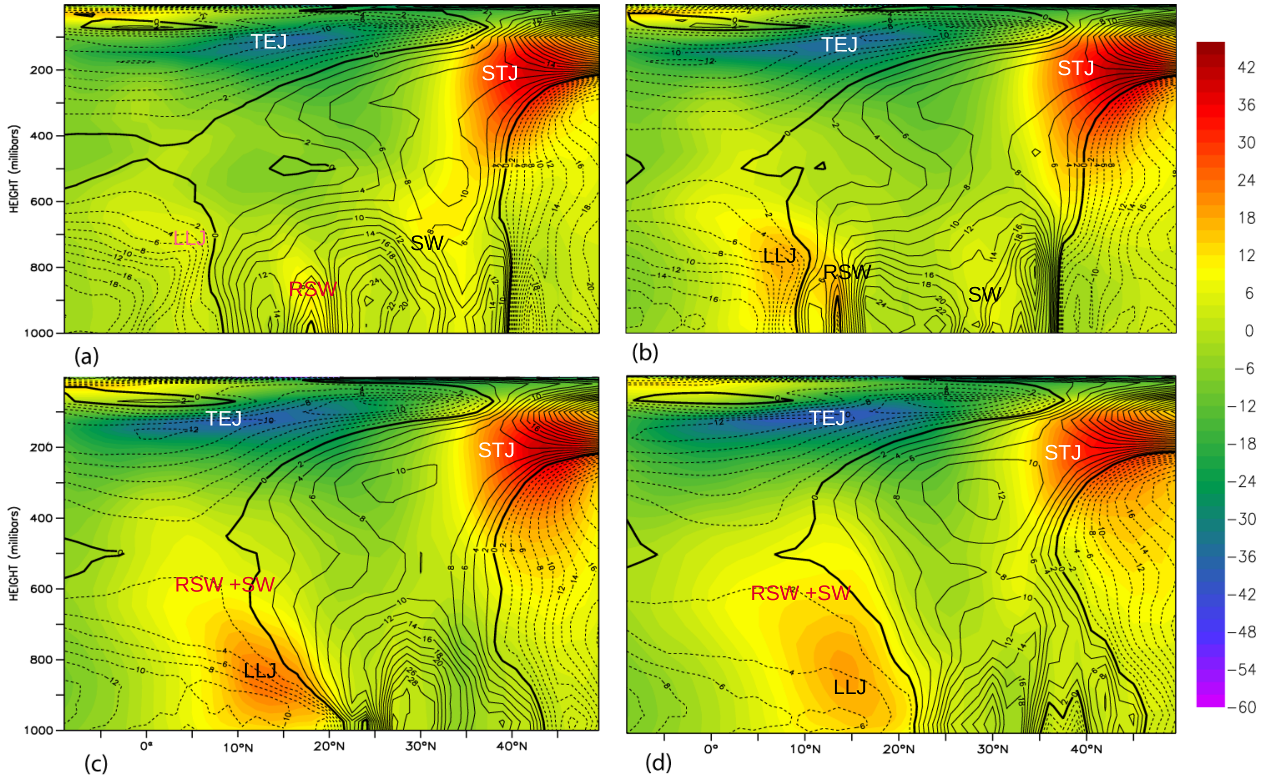

Supplementary Information Figure 2. (a) Zonal winds (clour shaded) and virtual potential temperature anomaly (K, contoured)) for the month of July 2013 along (a) 40°E (b) 50°E, (c) 60°E and (d) 70°E. Negative potential temperature anomaly indicated maritime air and positive potential temp indicates continental dry air. The major winds namely Tropical Easterly Jet (TEJ), Subtropical jet (STJ), Red Sea Winds (RSW), Low Level Jet (LLJ) and Shamal Winds (SW) are indicated. (a) At 40°E only the RSW is seen close to the surface. (b) (b) At 50°E, The LLJ maritime airmass and combined Res Sea and Shamal wind come close to each other. (c) At 60°E, the combined SW and RSW overrides the LLJ (d) At 70°E, the dry continental air mass can be seen over-riding the humid maritime airmass. At 70°E, the two airmasses appear to merge.

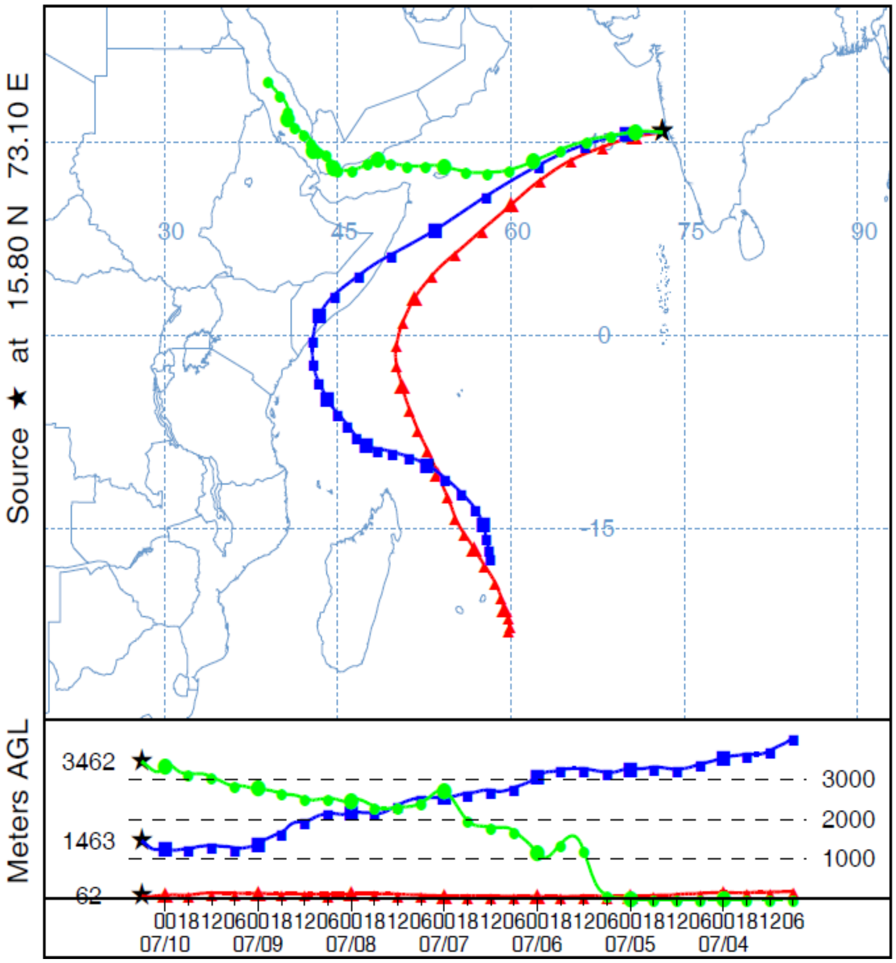

Supplementary Information Figure 3. Trajectory of winds back-tracked for 168 hrs using the HYSPLIT Model[1, 2]. The source location is Goa (15° 30' N; 73° 48' E) and start time 06:00 hrs UTC on 10 July 2013. The air mass below 1500 m over Goa is derivedmainly from the Arabian Sea and the Indian Ocean. Above 3000 m, the wind direction is variable but originating from semi-arid regions of N. Africa or the Middle-East. The uplift and overriding of the dusty continental winds (green line) over the maritime winds (blue and red lines) is clearly indicated in this model. Archieve trajectories were generated using the NOAA Air Resources Laboratory (ARL) HYSPLIT transport and dispersion model from the READY website (http://www.ready.noaa.gov). The authors gratefully acknowledge the NOAA Air

Resources Laboratory (ARL) for the provision of the HYSPLIT transport and dispersion model and/or READY website (http://www.ready.noaa.gov) used in this publication.

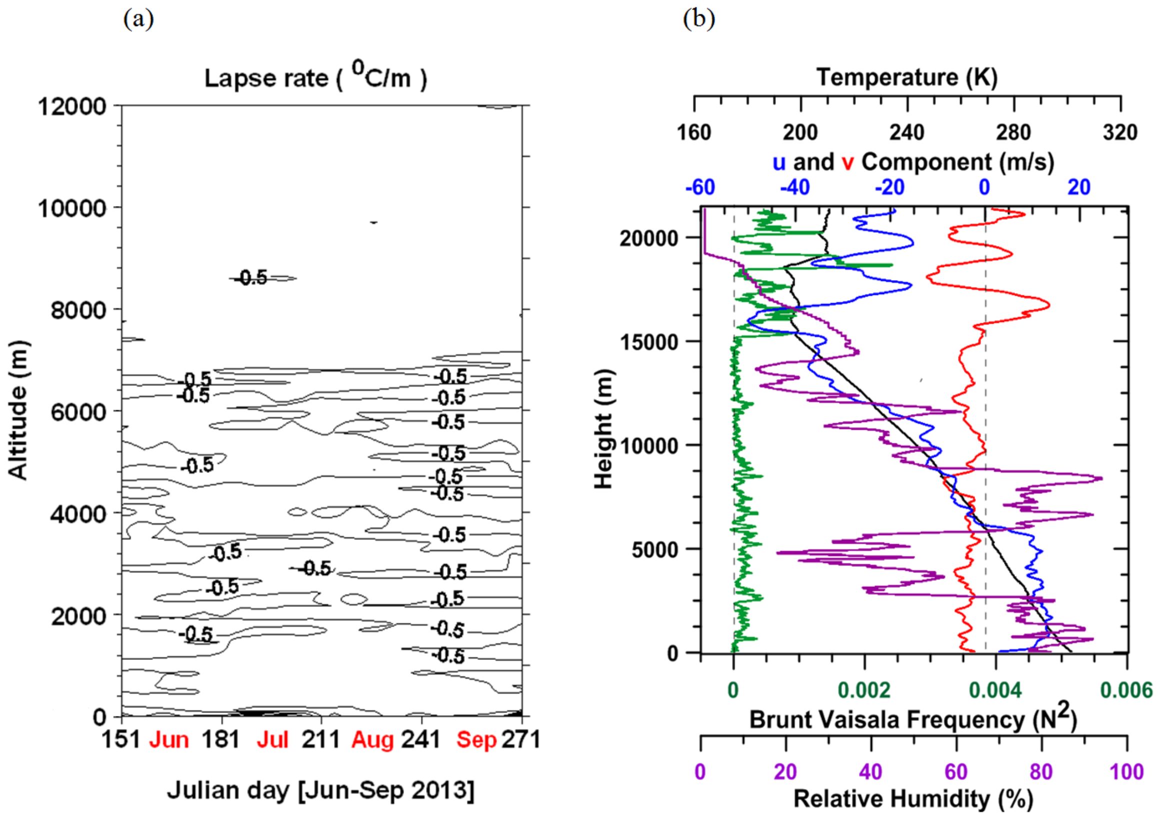

Supplementary Information Figure 4. (a) Time-series record of temperature lapse rate during summer monsoon months of the year 2013. Julian day from 1st June to 20th September is given in the X-axis and height (m) in the Y-axis. (b) Temperature, Relative Humidity, u & v component of the Wind Speed and Brunt Vaisala frequency (stability function) profiles at Goa during16 July 2013. The continental Shamal air mass (2 – 6 km), characterized by low humidity is present over a humid SW monsoon maritime air mass (0 – 2 km) derived from the Indian Ocean.

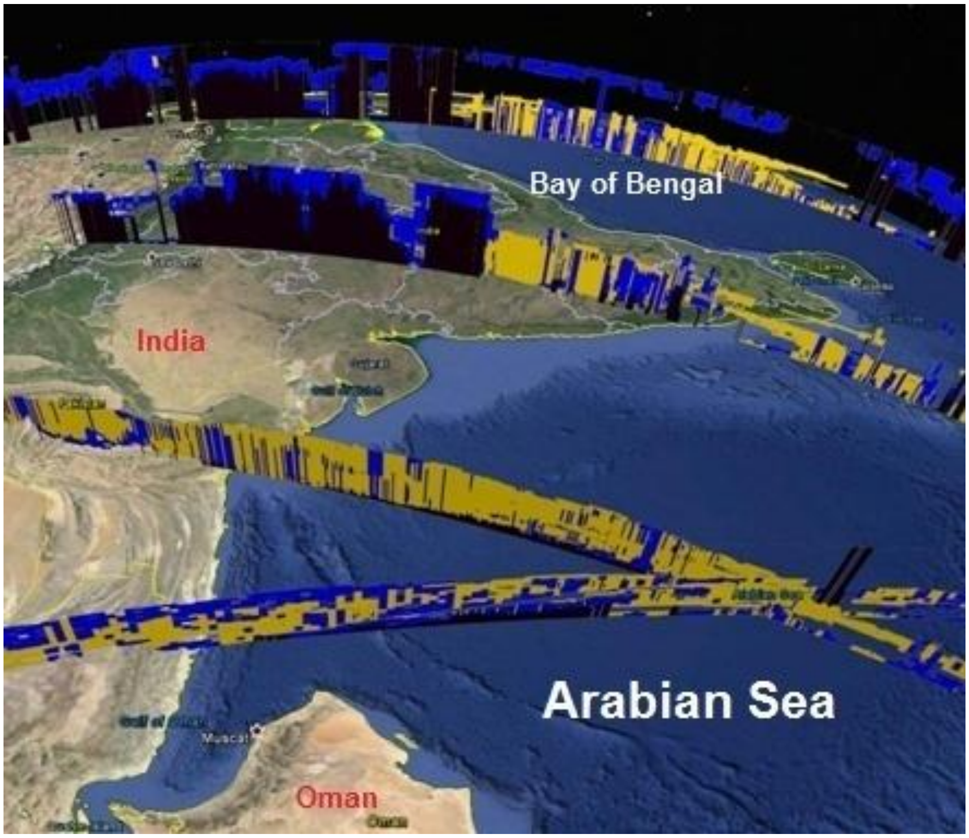

Supplementary Information Figure 5. Curtain plot of color-modulated, altitude-time image of Cloud-Aerosol Lidar and Infrared Pathfinder Satellite Observations (CALIPSO) 532 nm total attenuated backscatter (/km/sr) over the northern Indian Ocean for June 18, 2008. The height of curtain plot is 10 km. Yellow indicated dust, blue indicates clouds, and black no data. The plot shows dust layers extending up to a height of 5 km above sea level over the Arabian Sea, Indian Peninsula and reaching up to the central Bay of Bengal. CALIPSO data were obtained from the NASA Langley Research Center Atmospheric Science Data Center (https://www- calipso.larc.nasa.gov/products/lidar/browse_images/show_calendar.php.). KMZ file type images of CALIPSO's Expedited Lidar Level 1 Total_Attenuated_Backscatter_532 data were generated and final image generated using Google Earth Tool (https://earth.google.com/web/); Image @2017 Google, Image: Landsat/ Copernicus, US Department of State Geographer. Data Scripps Institute of Oceanography, NOAA, U.S. Navy, National Geospatial-Intelligence Agency, General Bathymetric Chart of the Oceans.

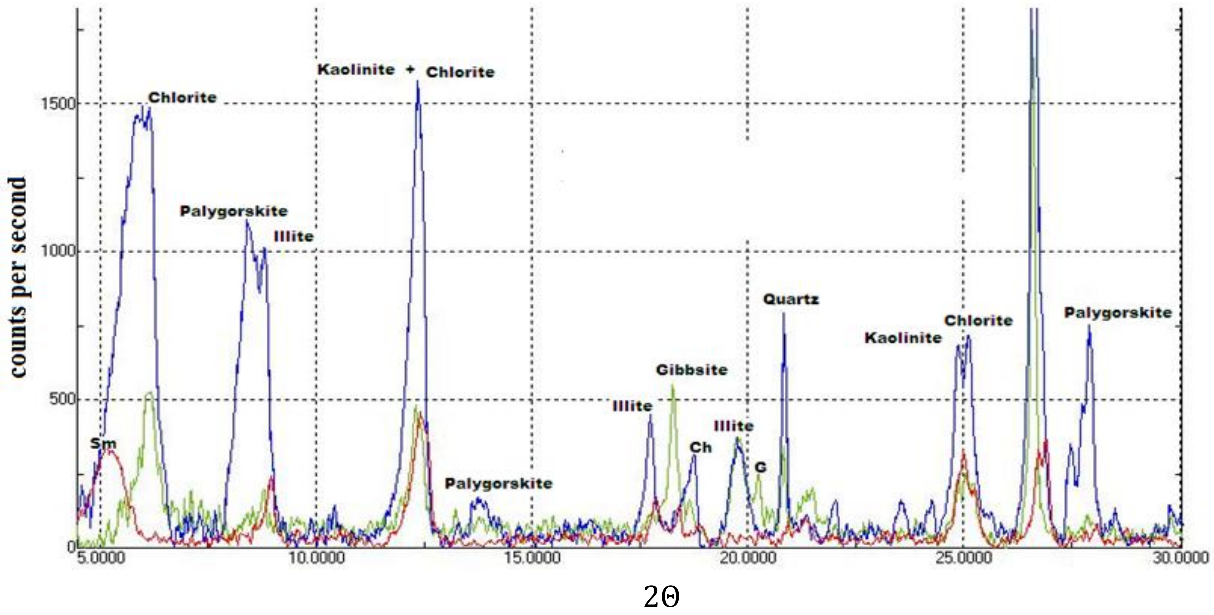

Supplementary Information Fig.6 . X-Ray diffractogram of rainwater particulate matter (blue line), suspended sediments from rivers of Goa (red) and local soil (green). The rainwater particles are distinctly more crystalline and contain higher amounts of palygorskite, illite, smectite and quartz compared to local crustal material having higher amounts of amorphous material and gibbsite.Ch, Sm and G are abbreviations for chlorite, smectite and gibbsite respectively

Oxide	Percentage
SiO_2	58.97
Al_2O_3	17.95
TiO_2	1.16
Fe_2O_3	9.91
MnO	0.07
MgO	6.57
CaO	1.84
Na_2O	0.75
K_2O	2.52
P_2O_5	0.26

Supplementary Information Table 1. Average concentration of major oxides in wet deposited material over Goa during June 2010 measured with X-Ray Fluorescence. The high concentration of SiO_2, $Al_2O_3$and TiO_2shows that the dominant material is mainlmineral dust from crustal sources.

Suplemantary information Table 2. Precipitation (mm/day) Wet Deposition of particulate matter (mg m^{-2} d^{-1}) and pH of rainwater for the year 2013. NM represents not measured.

Date	Precipitation	Wet Deposition	pH
28-May-13	0.10	NM	5.48
29-May-13	8.60	61.20	5.44
30-May-13	0.10	39.53	7.69
31-May-13	20.20	105.79	6.24
1-Jun-13	0.20	37.70	NM
2-Jun-13	15.70	37.70	NM
3-Jun-13	0.10	37.70	5.32
4-Jun-13	0.40	25.79	NM
5-Jun-13	5.70	8.46	5.26
6-Jun-13	46.00	25.54	5.65
7-Jun-13	40.70	169.58	5.65
8-Jun-13	94.80	169.58	6.07
9-Jun-13	50.10	169.58	8.14
10-Jun-13	33.90	169.58	6.74
11-Jun-13	0.10	494.58	8.98
12-Jun-13	37.20	434.66	8.42
13-Jun-13	0.60	219.45	8.36
14-Jun-13	16.40	609.44	NM
15-Jun-13	6.40	385.25	9
16-Jun-13	61.20	303.38	8.88
17-Jun-13	39.80	303.38	7.53
18-Jun-13	14.80	449.44	7.09
19-Jun-13	47.40	109.03	6.78
20-Jun-13	0.10	151.62	7.9
21-Jun-13	8.90	327.03	7.99
22-Jun-13	3.30	98.06	7.77
23-Jun-13	5.70	98.06	6.9
24-Jun-13	15.30	98.06	6.89
25-Jun-13	3.80	110.55	7.11
26-Jun-13	4.60	195.30	7.05
27-Jun-13	5.20	93.66	7.22
28-Jun-13	16.40	176.37	7.42
29-Jun-13	35.50	165.87	6.92
30-Jun-13	47.40	165.87	6.68
1-Jul-13	116.20	382.03	8.11
2-Jul-13	40.00	382.03	6.82

3-Jul-13	2.40	382.03	7
4-Jul-13	13.70	331.60	7.15
5-Jul-13	52.70	397.56	7.21
6-Jul-13	81.70	228.48	6.75
7-Jul-13	37.80	332.29	6.14
8-Jul-13	7.20	132.80	6.23
9-Jul-13	15.40	169.22	6.61
10-Jul-13	55.40	175.65	6.8
11-Jul-13	69.40	415.38	6.39
12-Jul-13	19.60	415.38	6.51
13-Jul-13	1.00	379.05	6.5
14-Jul-13	1.50	362.85	7.8
15-Jul-13	0.90	173.63	7.36
16-Jul-13	1.40	330.58	6.85
17-Jul-13	1.00	330.58	6.82
18-Jul-13	0.50	949.47	6.75
19-Jul-13	0.20	1018.70	6.72
20-Jul-13	0.00	506.53	6.57
21-Jul-13	0.10	415.94	6.87
22-Jul-13	39.20	496.76	7.38
23-Jul-13	64.50	721.40	7.1
24-Jul-13	29.70	847.80	7.2
25-Jul-13	35.10	569.26	7.16
26-Jul-13	48.20	248.82	6.54
27-Jul-13	16.10	279.83	9.07
28-Jul-13	8.50	635.74	6.85
29-Jul-13	13.50	193.93	6.5
30-Jul-13	12.40	266.60	7.1
31-Jul-13	16.10	142.76	8.03
1-Aug-13	3.00	519.15	7.79
2-Aug-13	2.80	87.78	7.75
3-Aug-13	4.00	130.11	7.34
4-Aug-13	1.10	130.11	7.69
5-Aug-13	11.70	117.12	7.3
6-Aug-13	2.10	70.19	7.22
7-Aug-13	1.50	124.24	6.99
8-Aug-13	1.90	19.67	6.89
9-Aug-13	4.50	92.53	6.69
10-Aug-13	1.60	50.37	6.6
11-Aug-13	7.80	29.85	6.8
12-Aug-13	18.60	NM	6.1

13-Aug-13	3.70	141.24	6.44
14-Aug-13	1.30	86.18	6.4
15-Aug-13	9.40	86.18	6.71
16-Aug-13	48.00	86.19	6.45
17-Aug-13	49.00	254.67	6.27
18-Aug-13	11.20	67.21	6.49
19-Aug-13	8.70	75.83	6.53
20-Aug-13	7.90	69.82	6.37
21-Aug-13	3.80	65.92	6.4
22-Aug-13	6.40	36.35	6.15
23-Aug-13	7.10	36.35	6.26
24-Aug-13	2.70	36.35	6.4
25-Aug-13	2.20	44.35	6.63
26-Aug-13	13.90	47.72	6.61
27-Aug-13	3.80	47.72	6.37
28-Aug-13	0.80	47.72	6.55
29-Aug-13	0.60	ND	5.84
30-Aug-13	0.10	67.78	6.77
31-Aug-13	0.00	NM	NM
1-Sep-13	0.00	NM	NM
2-Sep-13	0.00	NM	NM
3-Sep-13	1.90	NM	NM
4-Sep-13	0.40	NM	6.5
5-Sep-13	12.00	NM	5.13
6-Sep-13	2.40	63.22	6.04
7-Sep-13	0.20	14.38	NM
8-Sep-13	0.10	NM	NM
9-Sep-13	4.20	NM	NM
10-Sep-13	0.20	NM	4.77
11-Sep-13	0.50	42.30	NM
12-Sep-13	0.10	NM	5.07
13-Sep-13	0.00	NM	NM
14-Sep-13	0.00	NM	NM
15-Sep-13	1.60	NM	5.28
16-Sep-13	30.30	10.31	5.02
17-Sep-13	25.50	15.66	4.25
18-Sep-13	43.60	NM	5.07
19-Sep-13	16.60	15.87	4.76
20-Sep-13	18.70	127.15	5.75
21-Sep-13	13.50	218.76	6.34
22-Sep-13	6.70	50.21	5.97

23-Sep-13	11.10	33.85	5.69
24-Sep-13	6.60	21.21	6.4
25-Sep-13	11.50	10.67	4.81
26-Sep-13	2.10	20.15	5.04
27-Sep-13	0.20	9.03	6.44
28-Sep-13	13.80	95.14	5.89
29-Sep-13	19.00	95.14	NM
30-Sep-13	0.50	95.15	NM
1-Oct-13	0.30	NM	NM
2-Oct-13	0.00	NM	NM
3-Oct-13	31.30	NM	6.54
4-Oct-13	14.00	NM	5.65
5-Oct-13	0.20	NM	5.43
6-Oct-13	0.00	NM	NM
7-Oct-13	0.00	NM	NM
8-Oct-13	0.20	NM	NM
9-Oct-13	0.10	NM	NM
10-Oct-13	4.20	NM	5.4
11-Oct-13	0.50	25.85	5.04
12-Oct-13	6.00	20.86	4.83
13-Oct-13	7.30	9.06	4.93
14-Oct-13	0.20	7.54	4.62
15-Oct-13	0.20	10.66	NM
16-Oct-13	1.40	NM	5
17-Oct-13	1.50	NM	NM
18-Oct-13	0.10	NM	4.81
19-Oct-13	0.00	NM	NM
20-Oct-13	0.00	NM	NM
21-Oct-13	0.00	NM	NM
22-Oct-13	0.00	NM	NM
23-Oct-13	0.10	22.34	4.35

Suplementary information Table 3. Wet deposition of major ions in rainwater over Goa. All values are in millimoles m^{-2} day^{-1} . NSS-SO_4 is Non Sea Salt Sulphate. BD is below detection. Major ions were measured with a reagent free ion chromatograph. BD represents below detection levels.

Date	Na	NH_4	K	Mg	Ca	Cl	NO_3	SO_4	PO_4	NSS-SO_4
30-May-2013	2.86	0.19	0.12	0.44	1.93	3.54	0.58	0.62	0.00	0.45
31-May-2013	0.01	0.00	0.00	0.00	0.00	0.01	0.00	0.00	BD	0.00
3-Jun-2013	1.23	0.29	0.04	0.16	0.15	1.44	0.15	0.21	BD	0.13
4-Jun-2013	0.07	0.00	0.00	0.01	0.01	0.09	0.00	0.01	0.00	0.00
5-Jun-2013	0.07	0.01	0.00	0.01	0.01	0.08	0.01	0.01	0.00	0.01
6-Jun-2013	1.98	0.22	0.05	0.23	0.19	2.45	0.13	0.21	0.00	0.10
7-Jun-2013	2.14	0.34	0.04	0.32	0.47	2.45	0.25	0.42	BD	0.29
8-Jun-2013	4.67	0.18	0.09	0.59	2.23	5.65	0.31	0.57	0.00	0.28
10-Jun-2013	8.94	0.39	0.19	1.17	5.80	10.87	0.61	1.87	0.01	1.33
11-Jun-2013	27.10	0.04	0.72	3.22	14.61	33.92	1.20	5.47	0.01	3.84
14-Jun-2013	0.58	0.00	0.02	0.07	0.13	0.87	0.02	0.07	0.00	0.04
15-Jun-2013	7.41	0.07	0.18	0.90	2.79	11.94	0.32	1.14	0.00	0.69
16-Jun-2013	1.99	0.04	0.04	0.24	0.31	2.44	0.05	0.15	0.00	0.03
17-Jun-2013	5.65	0.48	0.11	0.79	2.14	9.60	0.67	1.14	0.01	0.80
18-Jun-2013	2.62	0.15	0.08	0.33	0.54	3.19	0.12	0.26	0.00	0.10
20-Jun-2013	6.19	0.81	0.15	0.81	3.01	8.06	0.87	1.34	0.01	0.96
22-Jun-2013	2.70	0.08	0.09	0.35	0.82	5.37	0.28	0.47	0.00	0.31
23-Jun-2013	0.93	0.03	0.02	0.11	0.18	1.29	0.05	0.11	0.00	0.05
25-Jun-2013	3.83	0.07	0.08	0.50	1.00	4.77	0.18	0.52	0.00	0.29
26-Jun-2013	2.06	0.04	0.06	0.27	0.56	2.51	0.13	0.20	0.00	0.08
27-Jun-2013	0.75	0.08	0.03	0.09	0.16	1.28	0.08	0.12	0.00	0.08
29-Jun-2013	1.38	0.59	0.03	0.20	0.29	1.98	0.17	0.26	0.00	0.17
30-Jun-2013	7.40	0.38	0.18	0.97	5.00	8.52	0.88	1.22	BD	0.78
1-Jul-2013	2.45	0.14	0.05	0.42	1.62	2.77	0.34	0.44	BD	0.29
2-Jul-2013	15.70	0.53	0.30	2.00	5.64	18.94	1.00	1.63	BD	0.69
3-Jul-2013	15.35	0.33	0.44	1.84	5.58	19.33	1.06	1.92	0.01	0.99
4-Jul-2013	0.58	0.02	0.01	0.07	0.22	0.69	0.05	0.07	BD	0.04
5-Jul-2013	1.69	0.16	0.04	0.24	0.80	1.97	0.20	0.24	0.00	0.14
6-Jul-2013	11.31	0.34	0.22	1.30	3.25	12.05	0.90	1.06	BD	0.38
7-Jul-2013	5.60	0.57	0.07	0.77	1.20	5.72	0.56	0.76	BD	0.42
8-Jul-2013	1.54	0.22	0.02	0.24	0.69	1.65	0.28	0.31	BD	0.22
9-Jul-2013	4.16	0.06	0.11	0.49	1.53	5.25	0.44	0.51	BD	0.26
10-Jul-2013	1.86	0.20	0.03	0.24	0.45	3.55	0.26	0.37	0.00	0.25

11-Jul-2013	10.25	0.31	0.23	1.24	2.14	9.92	0.59	0.88	0.01	0.26
12-Jul-2013	4.46	0.61	0.09	0.66	1.81	4.83	0.55	0.79	BD	0.52
13-Jul-2013	5.02	0.23	0.14	0.64	2.74	6.08	0.50	0.80	0.00	0.49
14-Jul-2013	0.12	0.01	0.01	0.02	0.11	0.16	0.02	0.02	BD	0.02
15-Jul-2013	0.23	0.01	0.01	0.03	0.13	0.18	0.02	0.03	BD	0.01
16-Jul-2013	0.17	0.01	0.00	0.02	0.06	0.18	0.01	0.02	BD	0.01
17-Jul-2013	0.38	0.01	0.01	0.04	0.14	0.30	0.02	0.03	BD	0.01
18-Jul-2013	0.21	0.01	0.00	0.03	0.08	0.18	0.01	0.02	0.00	0.01
19-Jul-2013	0.08	0.00	0.00	0.01	0.03	0.08	0.01	0.01	BD	0.00
20-Jul-2013	0.04	0.00	0.00	0.00	0.01	0.04	0.00	0.00	BD	0.00
21-Jul-2013	0.01	0.00	0.00	0.00	0.00	0.01	0.00	0.00	0.00	0.00
22-Jul-2013	0.02	0.00	0.00	0.00	0.01	0.02	0.00	0.00	BD	0.00
23-Jul-2013	2.91	0.42	0.05	0.39	0.95	3.35	0.29	0.44	BD	0.27
24-Jul-2013	12.59	0.66	0.27	1.61	4.91	16.78	1.04	2.04	0.00	1.28
25-Jul-2013	2.24	0.31	0.03	0.34	0.58	2.65	0.20	0.37	BD	0.23
26-Jul-2013	4.64	0.49	0.07	0.56	0.83	6.03	0.31	0.74	BD	0.46
27-Jul-2013	8.17	0.65	0.19	1.14	3.11	8.87	0.65	1.06	BD	0.57
28-Jul-2013	2.47	0.08	0.05	0.33	0.81	2.16	0.16	0.26	BD	0.11
29-Jul-2013	1.17	0.06	0.03	0.16	0.34	1.24	0.16	0.14	BD	0.07
30-Jul-2013	26.51	0.15	0.65	2.66	5.55	33.57	1.17	2.81	BD	1.22
31-Jul-2013	3.90	0.11	0.09	0.48	2.01	3.22	0.25	0.53	BD	0.30
1-Aug-2013	7.49	0.14	0.16	0.86	1.16	11.36	0.27	0.74	0.01	0.29
2-Aug-2013	1.65	0.02	0.04	0.20	0.46	2.33	0.09	0.17	0.00	0.08
3-Aug-2013	2.51	0.03	0.07	0.29	0.76	3.14	0.15	0.30	0.00	0.15
4-Aug-2013	1.44	0.03	0.04	0.19	0.65	1.83	0.10	0.22	0.00	0.13
5-Aug-2013	0.75	0.01	0.02	0.09	0.31	1.80	0.11	0.18	0.00	0.14
6-Aug-2013	1.49	0.05	0.03	0.21	1.06	1.90	0.16	0.25	0.01	0.16
7-Aug-2013	3.37	0.03	0.09	0.38	0.81	4.33	0.22	0.40	0.00	0.20
10-Aug-2013	1.48	0.05	0.07	0.18	0.55	1.61	0.12	0.16	BD	0.07
11-Aug-2013	1.68	0.01	0.05	0.20	0.38	2.21	0.14	0.20	0.00	0.09
12-Aug-2013	0.66	0.08	0.01	0.09	0.18	1.09	0.11	0.13	BD	0.09
13-Aug-2013	5.43	0.26	0.17	0.63	1.26	10.40	0.78	1.07	0.02	0.74
15-Aug-2013	0.09	0.01	0.00	0.01	0.03	0.17	0.02	0.03	0.00	0.02
16-Aug-2013	0.32	0.06	0.00	0.06	0.11	0.44	0.08	0.09	0.01	0.08
17-Aug-2013	0.47	0.02	0.00	0.15	0.14	0.69	0.07	0.09	BD	0.07
18-Aug-2013	4.75	0.42	0.08	0.65	1.19	6.26	0.53	0.76	0.04	0.48
19-Aug-2013	0.83	0.07	0.02	0.13	0.34	0.55	0.11	BD	BD	BD
20-Aug-2013	1.27	0.13	0.03	0.16	0.29	1.72	0.18	BD	0.01	BD
21-Aug-2013	2.18	0.14	0.07	0.25	0.32	1.90	0.13	BD	BD	BD
22-Aug-2013	0.37	0.02	0.01	0.05	0.07	0.35	0.05	BD	BD	BD

23-Aug-2013	0.33	0.06	0.01	0.05	0.09	0.43	0.06	BD	0.00	BD
24-Aug-2013	0.82	0.07	0.02	0.10	0.23	1.66	0.17	BD	BD	BD
25-Aug-2013	0.97	0.05	0.03	0.12	0.26	0.63	0.13	0.08	0.00	0.03
26-Aug-2013	0.13	0.03	0.00	0.02	0.04	0.14	0.02	BD	BD	BD
28-Aug-2013	0.31	0.04	0.02	0.04	0.08	0.21	0.06	0.03	0.00	0.01
29-Aug-2013	0.15	0.01	0.01	0.02	0.04	0.12	0.01	BD	0.00	BD
4-Sep-2013	0.28	0.02	0.01	0.04	0.10	0.19	0.03	0.03	BD	0.01
5-Sep-2013	0.00	0.00	0.00	0.00	0.00	0.00	0.00	0.00	BD	0.00
6-Sep-2013	0.65	0.32	0.03	0.10	0.11	0.44	0.08	0.08	BD	0.04
8-Sep-2013	0.18	0.01	0.01	0.02	0.05	0.13	0.02	0.01	0.00	0.00
10-Sep-2013	0.01	0.00	0.00	0.00	0.00	0.02	0.00	0.00	0.00	0.00
12-Sep-2013	0.13	0.02	0.01	0.01	0.01	0.14	0.04	0.02	0.00	0.01
15-Sep-2013	0.01	0.00	0.00	0.00	0.00	0.01	0.00	0.00	0.00	0.00
16-Sep-2013	0.02	0.01	0.00	0.01		0.02	0.01	0.00	BD	0.00
17-Sep-2013	0.42	0.33	0.01	0.11	0.12	0.54	0.42	0.24	BD	0.21
18-Sep-2013	0.57	0.20	0.01	0.10	0.09	0.88	0.27	0.14	BD	0.10
19-Sep-2013	0.64	0.36	0.01	0.17	0.17	0.60	0.54	0.26	BD	0.22
20-Sep-2013	0.34	0.28	0.01	0.09	0.28	0.49	0.40	0.21	BD	0.19
21-Sep-2013	0.25	0.05	0.00	0.07	0.09	0.32	0.88	0.06	BD	0.05
22-Sep-2013	3.60	0.40	0.11	0.39	0.44	4.32	1.10	0.46	BD	0.25
23-Sep-2013	1.85	0.16	0.05	0.22	0.25	1.25	0.12	0.13	0.00	0.02
24-Sep-2013	5.74	1.11	0.21	0.65	0.59	4.69	0.38	0.55	0.04	0.20
25-Sep-2013	1.35	0.15	0.06	0.18	0.12	0.46	0.04	0.05	0.01	-0.03
26-Sep-2013	1.71	0.21	0.14	0.20	0.13	0.57	0.05	0.07	0.02	-0.03
28-Sep-2013	0.15	0.01	0.01	0.02	0.05	0.17	0.03	0.03	0.00	0.02
29-Sep-2013	1.79	0.21	0.05	0.26	0.51	0.95	0.17	0.21	0.01	0.10
1-Oct-2013	0.48	0.00	0.02	0.06	0.14	0.51	0.07	0.07	0.00	0.04
3-Oct-2013	0.04	0.00	0.00	0.00	0.01	0.04	0.00	0.01	0.00	0.00
4-Oct-2013	2.65	0.76	0.05	0.36	0.28	1.48	0.16	0.23	BD	0.08
5-Oct-2013	5.11	1.02	0.48	0.52	0.39	2.14	0.25	0.23	0.02	-0.08
8-Oct-2013	0.33	0.03	0.03	0.04	0.08	0.35	0.10	0.06	0.00	0.04
10-Oct-2013	0.03	0.00	0.00	0.00	0.01	0.02	0.01	0.01	0.00	0.00
11-Oct-2013	0.19	0.04	0.02	0.04	0.05	0.09	0.05	0.03	0.00	0.02
12-Oct-2013	0.01	0.00	0.00	0.00	0.00	0.01	0.00	0.00	BD	0.00
13-Oct-2013	0.24	0.08	0.01	0.04	0.04	0.12	0.05	0.04	BD	0.03
16-Oct-2013	0.02	0.03	0.00	0.00	0.01	0.02	0.03	0.02	0.00	0.01

Supplementary information References

Stein, A.F., Draxler, R.R, Rolph, G.D., Stunder, B.J.B., Cohen, M.D., and Ngan, F., (2015). NOAA's HYSPLIT atmospheric transport and dispersion modeling system, Bull. Amer. Meteor. Soc., 96, 2059-2077, http://dx.doi.org/10.1175/BAMS-D-14-00110.1this link opens in a new window

Rolph, G., Stein, A., and Stunder, B., (2017). Real-time Environmental Applications and Display sYstem: READY. Environmental Modelling & Software, 95, 210-228, https://doi.org/10.1016/j.envsoft.2017.06.025this link opens in a new window. (http://www.sciencedirect.com/science/article/pii/S1364815217302360)this link opens in a new window
